# The place of new antibiotics for Gram-negative bacterial infections in intensive care: report of a consensus conference

**DOI:** 10.1186/s13613-023-01155-4

**Published:** 2023-07-04

**Authors:** Pierre-François Dequin, Cécile Aubron, Henri Faure, Denis Garot, Max Guillot, Olfa Hamzaoui, Virginie Lemiale, Julien Maizel, Joy Y. Mootien, David Osman, Marie Simon, Arnaud W. Thille, Christophe Vinsonneau, Khaldoun Kuteifan

**Affiliations:** 1 Inserm UMR 1100, Centre d’Etudes des Pathologies Respiratoires, Université, Tours, France; 2 Médecine Intensive Réanimation, Hôpital Bretonneau, 37044 Tours cedex 9 Tours, CHU France; 3 Médecine Intensive Réanimation CHU de Brest, Université de Bretagne Occidentale, Brest, France; 4 Médecine Intensive Réanimation, Centre Hospitalier Intercommunal Robert Ballanger, Aulnay Sous-Bois, France; 5 Médecine Intensive Réanimation CHU, Hôpital de Hautepierre, Strasbourg, France; 6 Médecine Intensive Réanimation CHU de Reims, Reims, France; 7 Medical ICU, Saint Louis Hospital, APHP, 1 Avenue Claude Vellefaux, Paris, France; 8 Medical Intensive Care Unit, Amiens University Hospital, Amiens, France; 9 Medical Intensive Care Unit, GHRMSA, Mulhouse, France; 10 Service de Médecine Intensive-Réanimation, AP-HP, Hôpital de Bicêtre, DMU CORREVE, Université Paris-Saclay, Le Kremlin-Bicêtre, France; 11 Maladies Infectieuses Et Tropicales, Hospices Civils de Lyon, Lyon, France; 12 Médecine Intensive Réanimation, Centre Hospitalier Universitaire de Poitiers, Université de Poitiers, Poitiers, France; 13 Service de Médecine Intensive Réanimation Centre Hospitalier de Bethune, Bethune, France

## Abstract

**Introduction:**

New beta-lactams, associated or not with beta-lactamase inhibitors (NBs/BIs), can respond to the spread of carbapenemase-producing enterobacteriales and nonfermenting carbapenem-resistant bacteria. The risk of emergence of resistance to these NBs/BIs makes guidelines necessary. The SRLF organized a consensus conference in December 2022.

**Methods:**

An ad hoc committee without any conflict of interest (CoI) with the subject identified the molecules (ceftolozane–tazobactam, ceftazidime–avibactam, imipenem–cilastatin–relebactam, meropenem–vaborbactam and cefiderocol); defined 6 generic questions; drew up a list of subquestions according to the population, intervention, comparison and outcomes (PICO) model; and reviewed the literature using predefined keywords. The quality of the data was assessed using the GRADE methodology. Seven experts in the field proposed their own answers to the questions in a public session and answered questions from the jury (a panel of 10 critical-care physicians without any CoI) and the public. The jury then met alone for 48 h to write its recommendations. Due to the frequent lack of powerful studies that have used clinically important criteria of judgment, the recommendations were formulated as expert opinions as often as necessary.

**Results:**

The jury provided 17 statements answering 6 questions: (1) Is there a place in the ICU for the probabilistic use of new NBs/IBs active against Gram-negative bacteria? (2) In the context of documented infections with sensitivity to several of these molecules, are there pharmacokinetic, pharmacodynamic, ecological or medico-economic elements for prioritization? (3) What are the possible combinations with these molecules and in what context? (4) Should we integrate these new molecules into a carbapenem-sparing strategy? (5) What pharmacokinetic and pharmacodynamic data are available to optimize their mode of administration in critically ill patients? (6) What are the dosage adaptations in cases of renal insufficiency, hepatocellular insufficiency or obesity?

**Conclusion:**

These recommendations should optimize the use of NBs/BIs in ICU patients.

**Supplementary Information:**

The online version contains supplementary material available at 10.1186/s13613-023-01155-4.

## Introduction

Bacterial ecology has changed in hospitals over the last few years with the emergence and spread of carbapenemase-producing Enterobacterales and nonfermenting bacteria that have developed resistance to carbapenem antibiotics, either through enzyme production or, more commonly, through altered permeability or efflux [1–6]. New antibiotics may help to control these germs, but they may also induce the emergence of resistant strains [7–11]. Published trials evaluating these antibiotics were generally noninferiority trials, and most did not target the resistant pathogens that are an issue in clinical practice. Some of these antibiotics have been presented as carbapenem-sparing [12], but the relevance of this concept needs to be discussed. Recently, Infectious Diseases Society of America [13] and European Society of Clinical Microbiology and Infectious Diseases [14] have published guidelines on similar topics, but not limited to the intensive care setting. The French Intensive Care Society (FICS, in French: Société de Réanimation de Langue Française, SRLF) organized a consensus conference on “the place of new antibiotics in Gram-negative bacterial infections in intensive care", as there was a need to define recommendations for the use of the new antibiotics available for critically ill patients, given the potentially low level of evidence in the available literature. It focused on newly available beta-lactam antibiotics, including two combinations of a cephalosporin with a beta-lactamase inhibitor (ceftolozane–tazobactam and ceftazidime–avibactam), two combinations of a carbapenem with a beta-lactamase inhibitor (imipenem–cilastatin–relebactam and meropenem–vaborbactam), and a fifth-generation cephalosporin (cefiderocol). Throughout this text, these new antibiotics will be grouped under the abbreviation NBs/BIs (new beta-lactams combined or not with beta-lactamase inhibitors).

## Methods

The SRLF appointed its Reference and Evaluation Committee to organize a consensus conference to better define the indications and conditions of use for these new antibiotics. The members of the committee defined six generic questions (Table 1), and then Patient, Intervention, Control, Outcome (PICO) questions were submitted to experts (Additional file 1) [15]. One expert was appointed for each generic question asked. A group of intensive care fellows and members of the committee carried out the bibliographic research in PubMed (contributors are listed in Additional file 1). Keywords were defined based on PICO questions. Grading of Recommendations Assessment, Development and Evaluation (GRADE) tables of published data were drawn up [16]. The level of evidence was assessed according to the type of study for each of the quoted references and then reassessed (increased or decreased) according to the quality of the study’s methodology. References were grouped according to each judging criterion. An overall quality of evidence was determined for each judging criterion based on the quality of evidence of each individual reference, the coherency of results between the different studies, whether the evidence was direct or indirect, and cost analysis. A “high” quality of evidence led to a “strong” recommendation (must, must not… GRADE 1 + or 1-). A moderate, low or very low quality of evidence led to an “optional” recommendation (probably should, probably should not… GRADE 2 + or 2-). In the absence of evidence, the issue was recommended in the form of an expert opinion.

**Table 1:** Questions put to the conference experts and panel

Question 1: Is there a place for the empirical use of the new beta-lactams active against Gram-negative bacteria in the intensive care setting?
Question 2: In the context of documented infections with susceptibility to more than one of these antibiotics, is there any pharmacokinetic, pharmacodynamic, ecological, or cost-effectiveness evidence for priorization?
Question 3: What are the possible combinations with these antibiotics, and in what context?
Question 4: Should these new antibiotics be included in a carbapenem-sparing strategy?
Question 5: What pharmacokinetic and pharmacodynamic data are available in critically ill patients to optimize the mode of administration, particularly continuous infusion, dose increase, and administration strategy guided by measurement of plasma antibiotic concentration?
Question 6: How should doses be adjusted in renal or hepatocellular failure or obesity?

The panel was made up of 10 members coordinated by a chairperson. All practiced or had practiced in intensive care, and two were also qualified in infectious diseases. They were chosen by the organizers on the one hand for their clinical interest in the topic, but on the other because they had no related potential conflicts of interest. At the end of the conference, the role of the panel was to provide a consensus text with the conclusions and recommendations of the conference in the form of a clear answer to each of the questions. The experts wrote a text for the panel members debating the assigned question, including the most recent scientific data, their opinions and arguments. A meeting was held for the experts, the panel members and a large audience of intensive care physicians. The experts presented their analyses and the specific scientific data for the question they were responsible for, and they answered the questions and comments of the panel and the public. After the public meeting, the panel met privately to draft the text answering the questions. Recommendations were formulated according to GRADE methodology. The proposed recommendations were presented and discussed individually. The aim was not necessarily to obtain a convergent opinion of the panel members for all of the proposals but to uncover points of agreement and points of disagreement or indecision. Each recommendation was then assessed by each panel member and scored individually from 1 (totally disagree) to 9 (strongly agree). The panel score was defined using a GRADE grid [17]. To achieve a strong recommendation, at least 70% of the participants had to agree. If there was no strong agreement, recommendations were reworded and then rescored to achieve consensus. Two recommendations required rewriting and a second round of voting to reach consensus. The final text contains the conclusions and recommendations of the conference.

### Question 1

Is there a place for the empirical use of the new beta-lactams active against Gram-negativeGram bacteria in the intensive care setting?

#### *Recommendation 1A*

*These antibiotics should probably not be used empirically in critically ill patients (grade 2-, moderate quality of evidence, strong agreement)*

#### *Recommendation 1B*

*The panel suggests that the use of these antibiotics should only be considered in the exceptional case of septic shock occurring in a patient with known colonization by carbapenemase-producing Enterobacterales or Pseudomonas aeruginosa resistant to any antipyocyanic antibiotic or in the event of an outbreak of one of these bacterial infections (panel opinion, strong agreement).*

#### Arguments

These recommendations are supported by the following data: first, for carbapenem-susceptible bacteria, no randomized controlled trial has shown a superiority of NBs/BIs over meropenem or the best available treatment [18–22]; second, colonization by Gram-negative bacteria resistant to carbapenems due to the production of carbapenemase is currently exceptional. In France, ertapenem-resistant Enterobacterale isolates vary between 0.02 and 0.2% [23]. A 2019 study in 11 Parisian hospitals found that only 1.2% of patients were colonized with carbapenemase-producing Enterobacteriales [4]. In an intensive care setting, REA-REZO 2018 data showed that 14.4% of health care-associated infections were attributed to *Pseudomonas aeruginosa*, with 23.3% of carbapenem-resistant strains, without specifying the mechanism of resistance [24]. Moreover, *P. aeruginosa* often combines several mechanisms of resistance, including an efflux system or lack of permeability due to porin inactivation, mutations in the penicillin-binding protein, and the overproduction of natural cephalosporinase [25]. Beta-lactams other than NBs/BIs may be active against all of these mechanisms. Third, less than 10% of patients colonized with multidrug-resistant (MDR) bacteria will develop an infection due to these bacteria, and the absence of colonization by MDR bacteria is an excellent negative predictive factor for MDR bacterial infection [26–28]. Fourth, similar to other beta-lactams, NBs/BIs exert selection pressure. For example, exposure to ceftazidime–avibactam led to the emergence of 20% resistance in Enterobacterales [7], and exposure to ceftolozane–tazobactam led to 15–50% resistance in *P. aeruginosa* [8, 9]. After exposure to ceftolozane–tazobactam, cross-resistance to ceftazidime–avibactam has also been reported [9, 10]. Rapid emergence of resistance has been reported with cefiderocol and, in addition, has been associated with excess mortality of patients infected with *Acinetobacter baumannii* [11].

Thus, given the lack of superiority of NBs/BIs over carbapenems, the low risk of MDR bacterial infection in the absence of prior colonization or an ongoing epidemic, the risk of the emergence of resistance, and the need to keep these antibiotics as a last resort, the panel suggests that their empirical use should be reserved for exceptional situations combining septic shock and known colonization by either carbapenem-resistant bacteria or *P. aeruginosa* resistant to other antipyocyanic antibiotics or in the event of a local epidemic of one of these germs. There are no data to support empirical use of NBs/BIs in the sole presence of risk factors for MDR bacterial colonization. Prior carbapenem therapy is a risk factor for the selection of carbapenem resistance in *P. aeruginosa* but is not sufficient to support the empirical use of NBs/BIs.

In exceptional cases where empirical administration of one of these antibiotics has been initiated, it is imperative that this therapy be reassessed and reduced if possible. This assumes that bacteriology laboratories reduce the time needed to determine antibiotic susceptibility, and to test available antibiotics without incorporating a priori strategies for sparing certain molecules.

### Question 2

In the context of documented infections with susceptibility to more than one of these antibiotics, is there any pharmacokinetic, pharmacodynamic, ecological, or cost-effectiveness evidence for prioritization?

#### *Recommendation 2A*

*There is insufficient evidence to prioritize ceftazidime–avibactam, meropenem–vaborbactam or imipenem–cilastatin–relebactam for carbapenem-resistant Enterobacterales infections when strains are susceptible to these antibiotics. (no recommendations, insufficient quality of evidence, strong agreement).*

#### Arguments

All three antibiotics are active against class A carbapenem-resistant Enterobacterales (*e.g.,* KPC). Ceftazidime–avibactam is the only compound active against class D carbapenem-resistant Enterobacterales (*e.g.,* OXA-48). None of these three antibiotics are active against carbapenem-resistant Enterobacterales carrying metallo-β-lactamases (*i.e.,* NDM or VIM). The intrinsic susceptibility profiles of each molecule are summarized in Table 2.

**Table 2:**
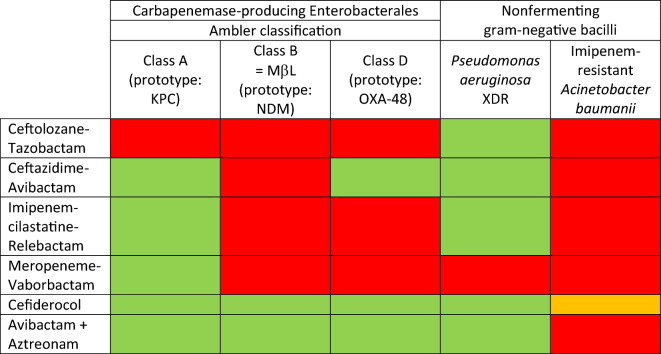
Spectrum of new beta-lactams with or without beta-lactamase inhibitors

KPC = *Klebsiella pneumoniae* carbapenemases; MβL = metallo-beta-lactamases; NDM = New-Delhi MβL; OXA-48 = oxacillinase-48; XDR = extensively drug-resistant [113]

Green boxes: mainly susceptible species

Red boxes: mainly resistant species

Orange box: despite being highly susceptible in vitro, clinical efficacy remains uncertain, with excess mortality in a subgroup of the credible trial [11]

No randomized controlled trial has compared these three new antibiotics in patients with carbapenem-resistant and non-carbapenem-resistant Enterobacterales infections. There are no pharmacokinetic, pharmacodynamic, ecological or cost-effectiveness arguments in favor of one of these antibiotics over the others if they are active in vitro.

Most randomized controlled trials assessing the efficacy of these three antibiotics did not target carbapenem-resistant Enterobacterales and most often used a carbapenem as a comparator [20, 21, 29–33]. Only two small randomized controlled trials specifically included patients infected with carbapenem-resistant Enterobacterales [34, 35].

Only one retrospective study compared ceftazidime–avibactam and meropenem–vaborbactam for carbapenem–resistant Enterobacterales infections. This study found no significant difference in mortality, clinical success at 30 and 90 days, or adverse events [36]. In this study, strains from patients receiving ceftazidime–avibactam developed resistance more often than those from patients receiving meropenem–vaborbactam, but not significantly so.

Several single-center or multicenter observational cohort studies have reported the efficacy of ceftazidime–avibactam or meropenem–vaborbactam, alone or in combination, for severe carbapenem-resistant Enterobacterales infections [37–48]. However, there are no published clinical data on the efficacy of imipenem–cilastatin–relebactam for KPC-producing Enterobacterales infections.

The size of the bacterial inoculum may impact the in vitro activity of these new antibiotics on carbapenem-resistant Enterobacterales [49], but the clinical significance remains unknown.

##### *Recommendation 2B*

*The panel suggests that cefiderocol should be used only if other therapies have failed (or are poorly tolerated) in documented infections with class A or D carbapenememase-producing Enterobacterales (panel opinion, strong agreement).*

#### Arguments

Cefiderocol is effective against class A or D carbapenemase-producing Enterobacterales and is the only antibiotic also effective against metallo-β-lactamase-producing Enterobacterales.

Clinical data on the efficacy of cefiderocol against carbapenem-resistant Enterobacterales infections are limited [11, 35, 50, 51]. An increase in the minimum inhibitory concentration of cefiderocol was reported in 15% of patients treated in the cefiderocol arm of the CREDIBLE-CR trial [11, 50].

Given the risk of emergence of cefiderocol-resistant strains, even though it is the only NB/BI with activity against metallo-β-lactamase-producing Enterobacterales, the panel did not recommend cefiderocol as first-line therapy.

##### *Recommendation 2C*

*There is insufficient evidence to prioritize cefiderocol over the combination of ceftazidime–avibactam plus aztreonam in documented infections with metallo-β-lactamase-producing Enterobacterales, especially NDM-producing strains (no recommendations, insufficient quality of evidence, strong agreement).*

#### Arguments

Cefiderocol is intrinsically active against metallo-β-lactamase-producing Enterobacterales. Another option for treating these infections is a combination of avibactam and aztreonam [52] (Table 2). Aztreonam is a monobactam that is not hydrolyzed by class B metallo-β-lactamases. However, it is hydrolyzed by the majority of other beta-lactamases, including KPC and AmpC. Additionally, most metallo-β-lactamase-producing Enterobacterales also produce other enzymes, notably class A serine-β-lactamases. Avibactam restores the activity of aztreonam against most carbapenem-resistant Enterobacterales. Thus, the combination of aztreonam and avibactam is active against bacteria that are resistant to either of these antibiotics individually [53]. No aztreonam–avibactam combination is currently marketed in France; therefore, aztreonam must be combined with ceftazidime–avibactam.

Available data suggest that these two options (cefiderocol and aztreonam–avibactam) are more efficient and cause less kidney injury than the use of older antibiotics [54, 55]. However, no comparison is currently available between these two options.

##### *Recommendation 2D*

*There is insufficient evidence to prioritize ceftolozane–tazobactam, ceftazidime–avibactam, and imipenem–cilastatin–relebactam in documented infections caused by Pseudomonas aeruginosa resistant to other antibiotics (no recommendations, insufficient quality of evidence, strong agreement).*

#### Arguments

In infections caused by *P. aeruginosa* resistant to carbapenems and other usually active beta-lactams (piperacillin–tazobactam, ceftazidime, cefepime, aztreonam), there are currently no randomized controlled trials or pharmacodynamic, pharmacokinetic, clinical, ecological, or cost-effectiveness arguments in the literature to favor one NB/BI over another in this indication.

It should be noted that meropenem–vaborbactam is intrinsically inactive against meropenem-resistant strains of *P. aeruginosa*.

Defining the minimum inhibitory concentrations (MICs) of *P. aeruginosa* could help in selecting the most appropriate NB/BI. Unfortunately, molecular diagnostic methods for the rapid identification of antibiotic resistance of *P. aeruginosa* do not allow for a definitive determination of which molecule to use, as this pathogen often has multiple resistance mechanisms. In the APECT-NP trial, the emergence of *P. aeruginosa* strains resistant by enzymatic mechanisms was comparable in the ceftolozane–tazobactam and meropenem groups; however, in the meropenem group, more second infections occurred with a different strain, resistant by mutation through efflux mechanisms [56].

##### *Recommendation 2E*

*The panel suggests that cefiderocol be used only in cases of treatment failure or intolerance to other therapies, for documented infections with Pseudomonas aeruginosa resistant to other antibiotics (panel opinion, strong agreement).*

#### Arguments

Cefiderocol is active against over 90% of carbapenem-resistant strains of *P. aeruginosa*, including strains resistant to the other three NBs/BIs mentioned above [57–60]. In a post hoc analysis of the CREDIBLE-CR trial, an increase in the MIC of cefiderocol was observed in 15% of patients receiving cefiderocol, without exceeding the susceptibility threshold of the molecule [11].

To preserve the efficacy of cefiderocol while minimizing the risk of emergence of resistant strains, the panel recommends limiting its use to cases for which there is no other alternative.

##### *Recommendation 2F*

*Cefiderocol should probably not be used for documented infections caused by carbapenem-resistant Acinetobacter baumannii unless there are no other treatment options available (grade 2-, moderate quality of evidence, strong agreement).*

#### Arguments

Despite its in vitro activity against the majority of carbapenem-resistant strains of *Acinetobacter baumannii*, currently available clinical data do not support the efficacy of cefiderocol for this indication. In the cefiderocol arm of the CREDIBLE-CR trial, the majority of deaths attributed to treatment failure occurred in patients with *A. baumannii* infections [11]. These results could be due to a phenomenon of heteroresistance to cefiderocol [61].

The treatment of severe infections due to carbapenem-resistant *A. baumannii* involves a combination of antibiotics, including colistin, aminoglycosides, tigecycline, and ampicillin–sulbactam, depending on the susceptibility profile of the strain, the site of infection, and the characteristics of the patient, after consultation with an infectious disease specialist. Ceftazidime–avibactam, meropenem–vaborbactam, imipenem–cilastatin–relebactam, and ceftolozane–tazobactam are not active against this pathogen.

### Question 3

What are the possible combinations with these antibiotics, and in what context?

#### *Recommendation 3*

*There is insufficient evidence to recommend combining these antibiotics with aminoglycosides or any other antibiotics (no recommendations, insufficient quality of evidence, strong agreement).*

#### Arguments

In vitro, synergy appears to exist between ceftazidime–avibactam or ceftolozane–tazobactam and aminoglycosides [62–68]. There are no available clinical data showing a benefit of these combinations, particularly with regard to survival [68–72]. As with older beta-lactams, a few clinical cases and retrospective studies have tested NBs/BIs combined with an aminoglycoside to broaden the antibiotic spectrum. These studies, of insufficient quality of evidence, did not show increased toxicity or any particularity compared to the combination of aminoglycosides with other beta-lactams.

Regarding colistin, the synergistic effect is variable in vitro [73–83]. The combination of ceftazidime–avibactam and colistin may have an antagonistic effect in vitro [79, 84]. Only in vivo cases emphasize the known nephrotoxicity of colistin [80].

For tigecycline [65, 74, 75, 85] and fosfomycin [77, 86, 87], synergy has been inconsistently observed in vitro. The clinical data are of very poor quality [69, 88].

### Question 4

Should these new antibiotics be included in a carbapenem-sparing strategy?

#### *Recommendation 4*

*The panel suggests that these new antibiotics should not be used as a part of a carbapenem-sparing strategy (panel opinion, strong agreement)*

#### Arguments

See the arguments in question 1 for recommendations on exceptional empirical use of NBs/BIs.

For documented infections with carbapenem- and NB/BI-susceptible pathogens (but those resistant to older beta-lactams), the panel’s recommendations are based on 4 arguments: first, with the exception of an ancillary study of a pivotal trial on ventilated-associated pneumonia [55], all the trials were in favor of simple noninferiority of NBs/BIs compared with carbapenems for Gram-negative infections sensitive to both types of antibiotics [18–21, 29]; second, health care cost data did not support the promotion of one class of antibiotics or the other [89]; third, the ecological impact of these antibiotics has not been fully assessed because their use is recent. However, some clinical trials have shown a rapid emergence of cross-resistance following the use of ceftolozane–tazobactam [8, 10], affecting up to 14% of *P. aeruginosa* strains. The emergence of resistance following exposure to ceftazidime–avibactam for infections with carbapenem-resistant Enterobacterales has been demonstrated in two clinical trials and has involved up to 15% of *Klebsiella pneumoniae* strains [38, 79]. A rapid increase in the MIC has been reported during treatment with cefiderocol [11]. All these data raise the fear of a rapid emergence of resistance to NBs/BIs if their use is not restricted. Fourth, these antibiotics appear to be the only antibiotics of last resort for infections with carbapenemase-producing Enterobacterales or with *P. aeruginosa* resistant to other antipyocyanic antibiotics.

Under these conditions, the panel considers it essential to preserve the use of NBs/BIs.

### Question 5

What pharmacokinetic and pharmacodynamic data are available in critically ill patients to optimize the mode of administration, particularly continuous infusion, dose increase, and administration strategy guided by measurement of plasma antibiotic concentration?

#### *Recommendation 5A*

*To increase the time that plasma levels of the antibiotic exceed the target concentration, these antibiotics should be administered as a prolonged infusion (2 to 4 h) (grade 1 + ,high quality of evidence, strong agreement).*

#### *Recommendation 5B*

*The panel suggests increasing the dose of some of these antibiotics in situations where there is a risk of underdosing, including increased renal clearance, high body mass index, and potentially difficult-to-reach infection sites (panel opinion, strong agreement).*

#### *Recommendation 5C*

*There is no evidence for routine plasma monitoring to guide the use of these antibiotics (no recommendations, insufficient quality of evidence, strong agreement).*

#### Arguments

To use beta-lactams properly, their mode of administration must be adapted to increase exposure to the antibiotic (percentage of time spent above the target concentration, calculated according to the MIC). Prolonged infusion and continuous infusion following a bolus are two modes that increase this exposure and have been shown to be superior, in pharmacokinetic and pharmacodynamic terms, to nonprolonged intermittent infusion [90–93]. No randomized clinical trial has shown the superiority of continuous infusion over prolonged infusion. In contrast, under some circumstances (high body mass index, hard-to-reach tissues, high volume of distribution, and increased renal clearance), plasma levels could, with continuous infusion, be permanently stabilized at an insufficient concentration [93]. Intermittent administration by prolonged infusion theoretically reduces this risk since each new injection generates a peak plasma concentration, thus avoiding the risk of permanent underdosing.

The use of a prolonged infusion optimizes the time during which the plasma concentrations of NBs/BIs are above the MIC [94–98]. The duration of prolonged infusion should be adapted to the stability of the molecule. All NBs/BIs except imipenem–cilastatin–relebactam are stable at 25 °C for more than 4 h, and 4-h infusions are recommended [99, 100]. For imipenem–cilastatin–relebactam, 3-h infusions ensure its stability at 25 °C [100].

As with other beta-lactams, certain clinical situations carry a risk of NB/BI underdosing: high body mass index, hard-to-reach infection site (especially the lungs, central nervous system, bones and joints), and high renal clearance (creatinine clearance > 130 ml/min/1.73 m^2^) [101, 102]. The marketing authorizations for certain antibiotics already provide an increased dosage for pulmonary infections. In other situations where there is a risk of NB/BI underdosing, increasing the daily dose of some of these antibiotics increases the time of exposure of the pathogen to an effective dose of the drug [103].

The assay of these different antibiotics is not available in all hospitals; for drugs combining a beta-lactam and a beta-lactamase inhibitor, the assay of the latter is not systematically available. The time required to obtain results may be long and not well suited to real-time drug administration. In addition, an NB/BI has a high therapeutic index. For these reasons, the panel did not select routine plasma monitoring to tailor NB/BI dosing.

If it is possible to obtain the results in time to adapt doses, it could be interesting, in situations where there is a particular risk of underdosing, to confront the residual plasma concentration of the molecule of interest with the MIC for the pathogen.

### Question 6

How should doses be adjusted in renal or hepatocellular failure or obesity?

#### *Recommendation 6A*

*In acute kidney injury, dosing should probably not be adjusted within the first 24 h of treatment (grade 2-, moderate quality of evidence, strong agreement).*

#### *Recommendation 6B*

*After the first 24 h of treatment, the dosage of these antibiotics should be adjusted according to the creatinine clearance or renal replacement therapy modalities if appropriate (grade 1 + , high quality of evidence, strong agreement).*

#### Arguments

Beta-lactams have a high therapeutic index, which means that the risk of antibiotic toxicity is limited compared to the risk of underdosing during the first days of a serious infection. Furthermore, in septic shock patients with acute kidney injury, the renal function usually improves, as shown in the trial by Gaudry et al., in which nearly 50% of patients had improved kidney function by the 72nd hour [104]. Thus, early dose adjustment of these antibiotics puts the patient at risk of underdosing in the first 24–48 h, justifying this delay before lowering the dose [105–107]. In one trial, the risk of emerging resistance to ceftazidime–avibactam was higher in cases with renal replacement therapy, possibly due to underdosing [108].

After 24-48 h, if the severity of kidney injury is confirmed, dose adjustment is warranted to avoid overdosing. This adjustment should be based on an estimate of the glomerular filtration rate by measuring the creatinine clearance and on the type of renal replacement therapy. Dose-adjustment depends on the molecule. In the case of intermittent hemodialysis, the dose of antibiotic must be injected again after each session to compensate for the elimination of the antibiotic during the session. In the case of continuous renal replacement therapy, dose-adjustment schedules are often imperfect because antibiotic elimination is correlated with the effluent flow rate, which varies frequently. Plasma monitoring seems relevant in this situation.

##### *Recommendation 6C*

*The panel suggests that doses of these antibiotics should not be decreased in patients with liver failure (panel opinion, strong agreement).*

#### Arguments

These antibiotics are all exclusively eliminated via the kidneys, without hepatic metabolism. Thus, impairment of liver function does not affect the elimination of these antibiotics. Therefore, there is no need to change the dosage of these antibiotics in case of impaired liver function. To our knowledge, no clinical trials have evaluated these antibiotics in patients with hepatocellular impairment.

##### *Recommendation 6D*

*The panel suggests that the dose of these antibiotics not be increased in obesity (panel opinion, strong agreement).*

#### Arguments

There are few published data on the administration of these antibiotics to obese patients. However, as with other beta-lactams, the hydrophilic nature of these antibiotics means that the change in volume of distribution is small in this population [109–111]. A trial using simulation (Monte Carlo model) for obese patients (body mass index between 35 and 65 kg/m^2^) treated with ceftolozane–tazobactam for complicated intra-abdominal or urinary tract infection achieved the target plasma concentration without requiring a change in the recommended dose schedule [112]*.*

Additional data are needed to refine the dosing schedule for this specific population.

## Supplementary Information

**Additional file 1.** members of the bibliography group; PICO questions (Patient, Intervention, Comparator, Outcome).

## Data Availability

Not applicable.

## References

[1] Jolivet S, Vaillant L, Poncin T, Gaudonnet Y, Rondinaud E, Bendjelloul G (2018). Prevalence of carriage of extended-spectrum β-lactamase-producing enterobacteria and associated factors in a French hospital. Clin Microbiol Infect.

[2] Pilmis B, Cattoir V, Lecointe D, Limelette A, Grall I, Mizrahi A (2018). Carriage of ESBL-producing *Enterobacteriaceae* in French hospitals: the PORTABLSE study. J Hosp Infect.

[3] Grall-Zahar I, Rucly S, Billard-Pomares T, Gasnier-Besnardeau K, Al Mouft O, Zahar JR, Zimhelt I (2022). Prevalence and risk factors for carriage of extended-spectrum β-lactamase-producing enterobacteriaceae in rehabilitation wards in France. Infect Dis Now.

[4] Grohs P, Vilfaillot A, Zahar JR, Barbut F, Frange P, Casetta A (2022). Faecal carriage of multidrug-resistant bacteria and associated risk factors: results from a point prevalence study. J Antimicrob Chemother.

[5] Lomont A, Sevin T, Assouvie L, Dalix A, Assoukpa J, Lecuru M, Lecointe D (2022). Carbapenemase-producing *Enterobacterales* and vancomycin-resistant *Enterococcus*
*faecium* carriage in patients who have traveled in foreign countries: a single center 5-year prospective study. Am J Infect Control.

[6] Macaux L, Ndoye O, Cordel H, Billard-Pomares T, Seytre D, Bouchaud O (2018). Extensively-drug-resistant bacteria carriers among overseas travellers: one-third had not been hospitalized previously. Int J Antimicrob Agents.

[7] Bush K, Bradford PA (2019). Interplay between beta-lactamases and new beta-lactamase inhibitors. Nat Rev Microbiol.

[8] Haidar G, Philips NJ, Shields RK, Snyder D, Cheng S, Potoski BA (2017). Ceftolozane-tazobactam for the treatment of multidrug-resistant *Pseudomonas*
*aeruginosa* infections: clinical effectiveness and evolution of resistance. Clin Infect Dis.

[9] Tamma PD, Beisken S, Bergman Y, Posch AE, Avdic E, Sharara SL, Cosgrove SE, Simner PJ (2021). Modifiable risk factors for the emergence of ceftolozane-tazobactam resistance. Clin Infect Dis.

[10] Rubio AM, Kline EG, Jones CE, Chen L, Kreiswirth BN, Nguyen MH (2021). In vitro susceptibility of multidrug-resistant *Pseudomonas*
*aeruginosa* following treatment-emergent resistance to ceftolozane-tazobactam. Antimicrob Agents Chemother.

[11] Bassetti M, Echols R, Matsunaga Y, Ariyasu M, Doi Y, Ferrer R (2021). Efficacy and safety of cefiderocol or best available therapy for the treatment of serious infections caused by carbapenem-resistant Gramnegative bacteria (CREDIBLE-CR): a randomised, open-label, multicentre, pathogen-focused, descriptive, phase 3 trial. Lancet Infect Dis.

[12] Bassetti M, Giacobe DR, Castaldo N, Russo A, Vena A (2021). Role of new antibiotics in extended-spectrum beta-lactamase- AmpC- infections. Curr Opin Infect Dis.

[13] Tamma PD, Aitken SL, Bonomo RA, Mathers AJ, van Duin D, Clancy CJ (2022). Infectious Diseases Society of America guidance on the treatment of extended-spectrum beta-lactamase producing *Enterobacterales* (ESBL-E), carbapenem-resistant *Enterobacterales* (CRE), and *Pseudomonas*
*aeruginosa* with difficult-to-treat resistance (DTR-*P*. *aeruginosa*). Clin Infect Dis.

[14] Paul M, Carrara E, Retamar P (2022). European Society of Clinical Microbiology and Infectious Diseases (ESCMID) guidelines for the treatment of infections caused by multidrug-resistant Gram-negative bacilli (endorsed by European Society of Intensive Care Medicine). Clin Microbiol Infect.

[15] Schardt C, Adams MB, Owens T, Keitz S, Fontelo P (2007). Utilization of the PICO framework to improve searching PubMed for clinical questions. BMC Med Inform Decis Mak.

[16] Guyatt G, Oxman AD, Akl EA, Kunz R, Vist G, Brozek J (2011). GRADE guidelines: 1. Introduction – GRADE evidence profiles and summary of finding tables. J Clin Epidemiol.

[17] Jaeschke R, Gyatt GH, Dellinger P, Schünemann H, Levy MM, Kunz R, Norris S, Bion J (2008). Use of GRADE grid to reach decisions on clinical practice guidelines is elusive. BMJ.

[18] Solomkin J, Hershberger E, Miller B, Popejoy M, Friedland I, Steenbergen J (2015). Ceftolozane-tazobactam plus metronidazole for complicated Intra-abdominal infections in an era of multidrug resistance: results from a randomized, double-blind, phase 3 trial (ASPECT-cIAI). Clin Infect Dis.

[19] Kollef MH, Nováček M, Kivistik Ü, Réa-Neto Á, Shime N, Martin-Loeches I (2019). Ceftolozane-tazobactam versus meropenem for treatment of nosocomial pneumonia (ASPECT-NP): a randomised, controlled, double-blind, phase 3, non-inferiority trial. Lancet Infect Dis.

[20] Wagenlehner FM, Sobel JD, Newell P, Armstrong J, Huang X, Stone GG (2016). Ceftazidime-avibactam versus doripenem for the treatment of complicated urinary tract infections, including acute pyelonephritis: RECAPTURE, a phase 3 randomized trial program. Clin Infect Dis.

[21] Torres A, Zhong N, Pachl J, Timsit JF, Kollef M, Chen Z (2018). Ceftazidime-avibactam versus meropenem in nosocomial pneumonia, including ventilator-associated pneumonia (REPROVE): a randomised, double-blind, phase 3 non-inferiority trial. Lancet Infect Dis.

[22] Torres A, Rank D, Melnick D, Rekeda L, Chen X, Riccobene T (2019). Randomized trial of ceftazidime-avibactam vs meropenem for treatment of hospital-acquired and ventilator-associated bacterial pneumonia (REPROVE): analyses per US FDA-specified end points. Open Forum Infect Dis.

[23] Surveillance de la résistance bactérienne aux antibiotiques en soins de ville et en établissements d'hébergement pour personnes âgées dépendantes. Mission Primo, résultats 2020. https://www.preventioninfection.fr/actualites/primo-surveillance-de-la-resistance-bacterienne-aux-antibiotiques-en-ville-et-en-ehpad-resultats-2020/

[24] REA-REZO Rapport annuel 2018 Infections associées aux soins en réanimation adulte. https://rearezo.chu-lyon.fr/resultats/rapport_rearezo_2018.pdf

[25] Plésiat P, Cattoir V, Bonnet R, Naas T, Dortet L, Centre National de Référence de la Résistance aux Antibiotiques. Rapport d’activité 2019-2020. https://www.cnr-resistance-antibiotiques.fr/ressources/pages/Rapport_CNR_RA_2019_2020v2.pdf

[26] Bruyere R, Vigneron C, Bador J, Aho S, Toitot A, Quenot JP (2016). Significance of prior digestive colonization with extended-spectrum beta-lactamase-producing *Enterobacteriaceae* in patients with ventilator-associated pneumonia. Crit Care Med.

[27] Houard M, Rouze A, Ledoux G, Six S, Jaillette E, Poissy J (2018). Relationship between digestive tract colonization and subsequent ventilator-associated pneumonia related to ESBL-producing *Enterobacteriaceae*. PLoS ONE.

[28] Kakoullis L, Papachristodoulou E, Chra P, Panos G (2021). Mechanisms of antibiotic resistance in important Gram-positive and Gram-negative pathogens and novel antibiotic solutions. Antibiotics (Basel).

[29] Carmeli Y, Aemstrong J, Laud PJ, Newell P, Stone G, Wardman A, Gasink LB (2016). Ceftazidime-avibactam or best available therapy in patients with ceftazidime-resistant *Enterobacteriaceae* and Pseudomonas aeruginosa complicated urinary tract infections or complicated intra-abdominal infections (REPRISE): a randomized, pathogen-directed, phase 3 study. Lancet Infect Dis.

[30] Mazuski JE, Gasink LB, Armstrong J, Broadhurst H, Stone GG, Rank D (2016). Efficacy and safety of ceftazidime-avibactam plus metronidazole versus meropenem in the treatment of complicated intra-abdominal infection: results from a randomized, controlled, double-blind, phase 3 program. Clin Infect Dis.

[31] Qin X, Tran BG, Kim MJ, Wang L, Nguyen DA, Chen Q (2017). A randomised, double-blind, phase 3 study comparing the efficacy and safety of Ceftazidime/avibactam plus metronidazole versus meropenem for complicated intra-abdominal infections in hospitalised adults in Asia. Int J Antimicrob Agents.

[32] Titov I, Wunderink RG, Roquilly A, Rodríguez Gonzalez D, David-Wang A, Boucher HW (2021). Randomized, double-blind, multicenter trial comparing efficacy and safety of imipenem-cilastatin-relebactam versus piperacillin-tazobactam in adults with hospital-acquired or ventilator-associated bacterial pneumonia (RESTORE-IMI 2 Study). Clin Infect Dis.

[33] Kaye KS, Bhowmick T, Metallidis S, Bleasdale SC, Sagan OS, Stus V (2018). Effect of meropenem-vaborbactam vs piperacillin-tazobactam on clinical cure or improvement and microbial eradication in complicated urinary tract infection: the TANGO I randomized clinical trial. JAMA.

[34] Motsch J, Murta de Oliveira C, Stus V, Köksal I, Lyulko O, Boucher HW (2020). RESTORE-IMI 1: a multicenter, randomized, double-blind trial comparing efficacy and safety of imipenem/relebactam vs. colistin plus imipenem in patients with imipenem-non-susceptible bacterial infections. Clin Infect Dis.

[35] Wunderink RG, Giamarellos-Bourboulis EJ, Rahav G, Mathers AJ, Bassetti M, Vazquez J (2018). Effect and safety of meropenem-vaborbactam versus best-available therapy in patients with carbapenem-resistant *Enterobacteriaceae* infections: the TANGO II randomized clinical trial. Infect Dis Ther.

[36] Ackley R, Roshdy D, Meredith J, Minor S, Anderson WE, Capraro GA, Polk C (2020). Meropenem-vaborbactam versus ceftazidime-avibactam for the treatment or carbapenem-resistant *Enterobacteriaceae*. Antimicrob Agents Chemother.

[37] Wilson GM, Fitzpatrick M, Walding K, Gonzalez B, Schweizer ML, Suda KJ, Evans CT (2021). Meta-analysis of clinical outcomes using ceftazidime/avibactam, ceftolozane/tazobactam, and meropenem/vaborbactam for the treatment of multidrug-resistant Gram-negative infections. Open Forum Infect Dis.

[38] Tumbarello M, Trecarichi EM, Corona A, De Rosa FG, Bassetti M, Mussini C (2019). Efficacy of ceftazidime-avibactam salvage therapy in patients with infections caused by *Klebsiella*
*pneumoniae* carbapenemase-producing *K*. *pneumoniae*. Clin Infect Dis.

[39] Tumbarello M, Raffaelli F, Cascio A, Falcone M, Signorini L, Mussini C (2022). Compassionate use of meropenem-vaborbactam for infections caused by KPC-producing *Klebsiella*
*pneumoniae*: a multicentre study. JAC Antimicrob Resist.

[40] Soriano A, Carmeli Y, Omrani AS, Moore LSP, Tawadrous M, Irani P (2021). Ceftazidime-avibactam for the treatment of serious Gram-negative infections with limited treatment options: a systematic literature review. Infect Dis Ther.

[41] Sousa A, Pérez-Rodríguez MT, Soto A, Rodríguez L, Pérez-Landeiro A, Martínez-Lamas L, Nodar A, Crespo M (2018). Effectiveness of ceftazidime/avibactam 31 as salvage therapy for treatment of infections due to OXA-48 carbapenemase producing *Enterobacteriaceae*. J Antimicrob Chemother.

[42] Shields RK, Nguyen MH, Chen L, Press EG, Potoski BA, Marini RV (2017). Ceftazidime-avibactam is superior to other treatment 30 regimens against carbapenem-resistant *Klebsiella*
*pneumoniae* bacteremia. Antimicrob Agents Chemother.

[43] Guimarães T, Nouér SA, Martins RCR, Perdigão Neto LV, Martins WMBS, Narciso Barbosa AC (2019). Ceftazidime-avibactam as salvage therapy for infections caused by *Enterobacterales* coresistant to carbapenems and polymyxins. Antimicrob Agents Chemother.

[44] Karaiskos I, Daikos GL, Gkoufa A, Adamis G, Stefos A, Symbardi S (2021). Hellenic Ceftazidime/Avibactam Registry Study Group. Ceftazidime/avibactam in the era of carbapenemase-producing *Klebsiella*
*pneumoniae* experience from a national registry study. J Antimicrob Chemother.

[45] De la Calle C, Rodríguez O, Morata L, Marco F, Cardozo C, García-Vidal C (2019). Clinical characteristics and prognosis of infections caused by OXA-48 carbapenemase-producing *Enterobacteriaceae* in patients treated with ceftazidime-avibactam. Int J Antimicrob Agents.

[46] Castón JJ, Gallo M, García M, Cano A, Escribano A, Machuca I, Spanish Network for Research in Infectious Diseases (REIPI) (2020). Ceftazidime-avibactam in the treatment of infections caused by KPC-producing *Klebsiella*
*pneumoniae*: factors associated with clinical efficacy in a single-center cohort. Int J Antimicrob Agents.

[47] Alraddadi BM, Saeedi M, Qutub M, Alshukairi A, Hassanien A, Wali G (2019). Efficacy of ceftazidime-avibactam in the treatment of infections due to carbapenem-resistant *Enterobacteriaceae*. BMC Infect Dis.

[48] Alosaimy S, Lagnf AM, Morrisette T, Scipione MR, Zhao JJ, Jorgensen SCJ (2021). Real-world, multicenter experience with meropenem-vaborbactam for Gram-negative bacterial infections including carbapenem-resistant *Enterobacterales* and *Pseudomonas*
*aeruginosa*. Open Forum Infect Dis.

[49] Danjean M, Hobson CA, Gits-Muselli M, Courroux C, Monjault A, Bonacorsi S, Birgy A (2022). Evaluation of the inoculum effect of new antibiotics against carbapenem-resistant *Enterobacterales*. Clin Microbiol Infect.

[50] Bassel M, Echols R, Matsunaga Y, Ariyasu M, Doi Y, Ferrer R (2021). Efficacy and safety of cefiderocol or best available therapy for the treatment of serious infections caused by carbapenem-resistant Gram-negative bacteria (CREDIBLE-CR): a randomised, open-label, multicentre, pathogen-focused, descriptive, phase 3 trial. Lancet Infect Dis.

[51] Wunderink RG, Matsunaga Y, Ariyasu M, Clevenbergh P, Echols R, Kaye KS (2021). Cefiderocol versus high-dose, extended-infusion meropenem for the treatment of Gram-negative nosocomial pneumonia (APEKS-NP): a randomised, doubleblind, phase 3, non-inferiority trial. Lancet Infect Dis.

[52] Falcone M, Daikos GL, Tiseo G, Bassoulis D, Giordano C, Galfo V (2021). Efficacy of ceftazidime avibactam plus aztreonam in patients with bloodstream infections caused by metallo-beta-lactamases –producing *Enterobacterales*. Clin Inf Dis.

[53] Sader HS, Carvalhaes CG, Arends SJR, Castanheira M, Mendes RE (2021). Aztreonam-avibactam activity against clinical isolates of *Enterobacterales* collected in Europe, Asia and Latin America in 2019. J Antimicrob Chemother.

[54] Falcone M, Tiseo G, Leonildi A, Della Sala L, Vecchione A, Barnini S, Farcomeni A, Menichetti F (2018). Cefiderocol versus imipenem-cilastatin for the treatment of complicated urinary tract infections caused by Gram-negative uropathogens: a phase 2, randomised, double-blind, non-inferiority trial. Lancet Infect Dis.

[55] Timsit JF, Paul M, Shields RK, Echols R, Baba T, Yamano Y, Portsmouth S (2022). Cefiderocol for the treatment of infections due to metallo-beta-lactamase producing pathogens in the CREDIBLE-CR and APEKS-NP phase 3 randomized studies. Clin Infect Dis.

[56] Johnson MG, Bruno C, Castanheira M (2021). Evaluating the emergence of nonsusceptibility among *Pseudomonas*
*aeruginosa* respiratory isolates from a phase-3 clinical trial for treatment of nosocomial pneumonia ASPECT-NP). Intern J Antimicrob Agents.

[57] Hackel MA, Lomovskaya O, Dudley MN, Karlowsky JA, Sahm DF (2017). In vitro activity of meropenem-vaborbactam against clinical isolates pf KPC-positive *Enterobacteriaceae*. Antimicrob Agants Chemother..

[58] Ito A, Kohira N, Bouchillon SK, West J, Rittenhouse S, Sader HS (2016). In vitro antimicrobial activity of S-649266, a catechol-substituted siderophore cephalosporin, when tested against non-fermenting Gram-negative bacteria. J Antimicrob Chemother.

[59] Jacobs MR, Abdelhamed AM, Good CE, Rhoads DD, Hujer KM, Hujer AM (2018). ARGONAUT-I: activity of cedirocol (S-649266), a siderophore cephalosporin, against Gram-negative bacteria, including carbapenem-resistant nonfermenters and *Enterobacteriaceae* with defined extended-spectrum beta-lactamases and carbapenemases. Antimicrob Agents Chemother.

[60] Candel FJ, Henriksen AS, Longshaw C, Yamano Y, Oliver A (2022). In vitro activity of the novel siderophore cefiderocol, in Gram-negative pathogens in Europe by site of infection. Clin Microbiol Infect.

[61] Choby JE, Ozturk T, Satola SW, Jacob JT, Weiss DS (2021). Does cefiderocol heteroresistance explain the discrepancy between the APEKS-NP and CRDIBLE-CR clinical trial results?. Lancet Microbe.

[62] Almarzoky A, Safa S, Kuti JL, Nicolau DP (2018). Antibacterial activity of human simulated epithelial lining fluid concentrations of ceftazidime-avibactam alone or in combination with amikacin inhale (BAY41-6551) against carbapenem-resistant *Pseudomonas*
*aeruginosa* and *Klebsiella*
*pneumoniae*. Antimicrob Agents Chemother.

[63] Dassner AM, Sutherland C, Girotto J, Nicolau DP (2017). In vitro activity of ceftolozane-tazobactam alone or with an aminoglycoside against multi-drug-resistant *Pseudomonas*
*aeruginosa* from pediatric cystic fibrosis patients. Infect Dis Ther.

[64] Galani I, Papoutsaki V, Karantani I, Karaiskos I, Galani L, Adamou P (2020). In vitro activity of ceftolozane-tazobactam alone and in combination with amikacin against MDR/XDR *Pseudomonas*
*aeruginosa* isolates from Greece. J Antimicrob Chemother.

[65] Jacqueline C, Howland K, Chesnel L (2017). In vitro activity of ceftolozane-tazobactam in combination with other classes of antibacterial agents. J Glob Antimicrob Resist.

[66] Monogue ML, Abbo LM, Rosa R, Camargo JF, Martinez O, Bonomo RA, Nicolau dP.  (2017). In vitro Discordance with in vivo activity: humanized exposures of ceftazidime-avibactam, aztreonam, and tigecycline Alone and in combination against New Delhi Metallo-β-Lactamase-producing *Klebsiella*
*pneumoniae* in a murine lung infection model. Antimicrob Agents Chemother.

[67] Noel AR, Bowker KE, Attwood M, MacGowan AP (2018). Antibacterial effect of ceftolozane-tazobactam in combination with amikacin against aerobic Gram-negative bacilli studied in an in vitro Pharmacokinetic model of infection. J Antimicrob Chemother.

[68] Rico Caballero V, Almarzoky Abuhussain S, Kuti JL, Nicolau DP (2018). Efficacy of human-simulated exposures of ceftolozane-tazobactam alone and in combination with amikacin or colistin against multidrug-resistant *Pseudomonas*
*aeruginosa* in an in vitro pharmacodynamic model. Antimicrob Agents Chemother.

[69] Zheng G, Cai J, Zhang L, Chen D, Wang L, Qiu Y (2022). Ceftazidime-avibactam-based versus polymyxin B-based therapeutic regimens for the treatment of carbapenem-resistant *Klebsiella*
*pneumoniae* infection in critically Ill patients: a retrospective cohort study. Infect Dis Therap.

[70] Vickery SB, McClain D, Wargo KA (2016). Successful use of ceftolozane-tazobactam to treat a Pulmonary exacerbation of cystic fibrosis caused by multidrug-resistant *Pseudomonas*
*aeruginosa*. Pharmacother J Hum Pharmacol Drug Ther.

[71] Ottino L, Bartalesi F, Borchi B, Bresci S, Cavallo A, Baccani I, Rossolini GM, Bartoloni A (2021). Ceftolozane-tazobactam for *Pseudomonas*
*aeruginosa* pulmonary exacerbations in cystic fibrosis adult patients: a case series. Eur J Clin Microbiol Infect Dis.

[72] Pegh-Peghin M, Maiani M, Castaldo N, Givone F, Righi E, Lechiancole A (2018). Ceftolozane-tazobactam for the treatment of MDR *Pseudomonas*
*aeruginosa* left ventricular assist device infection as a bridge to heart transplant. Infection.

[73] Borjan J, Meyer KA, Shields RK, Wenzler E (2020). Activity of ceftazidime-avibactam alone and in combination with polymyxin B against carbapenem-resistant *Klebsiella*
*pneumoniae* in a tandem in vitro time-kill/in vivo Galleria mellonella survival model analysis. Internat J Antimicrob Agents.

[74] Mataraci-Kara E, Yilmaz M, Özbek-Çelik B (2019). In vitro activities of ceftazidime-avibactam alone or in combination with antibiotics against multidrug-resistant *Acinetobacter*
*baumannii* Isolates. J Global Antimicrob Resist.

[75] Mataraci-Kara E, Yilmaz M, Tosun AI, Özbek-Çelik B (2020). Evaluation of the synergy of ceftazidime-avibactam in combination with colistin, doripenem, levofloxacin, tigecycline, and tobramycin against OXA-48 producing enterobacterales. J Chemother.

[76] Mataraci-Kara E, Yilmaz M, Tosun AI, Özbek-Çelik B (2020). Synergistic activities of ceftazidime-avibactam in combination with different antibiotics against colistin-non-susceptible clinical strains of *Pseudomonas*
*aeruginosa*. Infect Dis.

[77] Mikhail S, Singh NB, Kebriaei R, Rice SA, Stamper KC, Castanheira M, Rybak MJ (2019). Evaluation of the synergy of ceftazidime-avibactam in combination with meropenem, amikacin, aztreonam, colistin, or fosfomycin against well-characterized multidrug-resistant *Klebsiella*
*pneumoniae* and *Pseudomonas*
*aeruginosa*. Antimicrob Agents Chemother.

[78] Montero MM, Ochoa SD, López-Causapé L, Luque S, Sorlí L, Campillo N, Montesinos IL (2021). Time-kill evaluation of antibiotic combinations containing ceftazidime-avibactam against extensively drug-resistant Pseudomonas aeruginosa and their potential role against ceftazidime-avibactam-resistant isolates. Microbiology Spectrum.

[79] Shields RK, Nguyen MH, Hao B, Kline EG, Clancy CJ (2018). Colistin does not potentiate ceftazidime-avibactam killing of carbapenem-resistant *Enterobacteriaceae* in vitro or suppress emergence of ceftazidime-avibactam resistance. Antimicrob Agents Chemother.

[80] Wang F, Zhou Q, Yang X, Bai Y, Cui J (2021). Evaluation of ceftazidime-avibactam alone and in combination with amikacin, colistin and tigecycline against *Klebsiella*
*pneumoniae* carbapenemase-producing *K*. *pneumoniae* by in vitro time- kill experiment. PLoS ONE.

[81] Gómez-Junyen J, Benavent E, Sierra Y, El Haj C, Soldevila L, Torrejón B (2019). Efficacy of ceftolozane-tazobactam alone and in combination with colistin against multidrug-resistant *Pseudomonas*
*aeruginosa* in an in vitro biofilm pharmacodynamic model. Internat J Antimicrob Agents.

[82] Asempa TE, Nicolau DP, Kuti JL (2019). In vitro activity of imipenem-relebactam alone or in combination with amikacin or colistin against *Pseudomonas*
*aeruginosa*. Antimicrob Agents Chemother.

[83] Chen T, Xu W, Yu K, Zeng W, Xu C, Cao J, Zhou T (2021). In vitro activity of ceftazidime-avibactam alone and in combination with amikacin against colistin-resistant Gram-negative pathogens. Microb Drug Resistance.

[84] Nath S, Moussavi F, Abraham D, Landman D, Quale J (2018). In vitro and in vivo activity of single and dual antimicrobial agents against KPC-producing *Klebsiella*
*pneumoniae*. J Antimicrob Chemother.

[85] Balabanian G, Rose M, Manning N, Landman D, Quale J (2018). Effect of porins and Bla _KPC_ expression on activity of imipenem with relebactam in *Klebsiella*
*pneumoniae*: can antibiotic combinations overcome resistance?. Microb Drug Resistance.

[86] Ojdana D, Gutowska A, Sacha P, Majewski P, PWieczorek P, Tryniszewska E.  (2019). Activity of ceftazidime-avibactam alone and in combination with ertapenem, fosfomycin, and tigecycline against carbapenemase-producing *Klebsiella*
*pneumoniae*. Microb Drug Resistance..

[87] Papp-Wallace KM, Zeiser ET, Becka SA, Park S, Wilson BM, Winkler ML, D’Souza R (2019). Ceftazidime-avibactam in combination with fosfomycin: a novel therapeutic strategy against multidrug-resistant *Pseudomonas*
*aeruginosa*. J Infect Dis.

[88] Zheng G, Zhang J, Wang B, Cai J, Wang L, Hou K (2021). Ceftazidime-avibactam in combination with in vitro non-susceptible antimicrobials versus ceftazidime-avibactam in monotherapy in critically ill patients with carbapenem-resistant *Klebsiella*
*pneumoniae* infection: a retrospective cohort study. Infect Dis Ther.

[89] Naik J, Puzniak L, Critchlow S, Elsea D, Dillon RJ, Yang J (2021). Cost effectiveness of ceftolozane-tazobactam compared with meropenem for the treatment of patients with ventilated hospital-acquired bacterial pneumonia and ventilator-associated bacterial pneumonia. Infect Dis Ther.

[90] Benko AS, Cappelletty DM, Kruse JA, Rybak MJ (1996). Continuous infusion versus intermittent administration of ceftazidime in critically ill patients with suspected gram-negative infections. Antimicrob Agents Chemother.

[91] Lodise TP, Lomaestro B, Drusano GL (2007). Piperacillin-tazobactam for *Pseudomonas*
*aeruginosa* infection: clinical implications of an extended-infusion dosing strategy. Clin Infect Dis.

[92] Rhodes NJ, Liu J, O’Donnell JN (2018). Prolonged infusion piperacillin-tazobactam decreases mortality and improves outcomes in severely ill patients: results of a systematic review and meta-analysis. Crit Care Med.

[93] Vardakas KZ, Voulgaris GL, Maliaros A (2018). Prolonged versus short-term intravenous infusion of antipseudomonal β-lactams for patients with sepsis: a systematic review and meta- analysis of randomised trials. Lancet Infect Dis.

[94] Dheyriat L, Bourguignon L, Perpoint T (2022). Pharmacokinetic/pharmacodynamic simulations of cost-effective dosage regimens of ceftolozane-tazobactam and ceftazidime-avibactam in patients with renal impairment. Antimicrob Agents Chemother.

[95] Sy SKB, Zhuang L, Sy S, Derendorf H (2019). Clinical pharmacokinetics and pharmacodynamics of ceftazidime-avibactam combination: a model-informed strategy for its clinical development. Clin Pharmacokinet.

[96] Matsumoto S, Singley CM, Hoover J (2019). Efficacy of cefiderocol against carbapenem-resistant Gram-negative bacilli in immunocompetent-rat respiratory tract infection models recreating human plasma pharmacokinetics. Antimicrob Agents Chemother.

[97] Couffignal C, Pajot O, Laouénan C (2014). Population pharmacokinetics of imipenem in critically ill patients with suspected ventilator-associated pneumonia and evaluation of dosage regimens. Br J Clin Pharmacol.

[98] Griffith DC, Sabet M, Tarazi Z (2019). Pharmacokinetics/pharmacodynamics of vaborbactam, a novel beta-lactamase inhibitor, in combination with meropenem. Antimicrob Agents Chemother.

[99] Loeuille G, D’Huart E, Vigneron J (2022). Stability studies of 16 antibiotics for continuous infusion in Intensive Care Units and for performing outpatient parenteral antimicrobial therapy. Antibioth Basel Switz.

[100] Viaene E, Chanteux H, Servais H (2002). Comparative stability studies of antipseudomonal β-lactams for potential administration through portable elastomeric pumps (home therapy for cystic fibrosis patients) and motor-operated syringes (Intensive Care Units). Antimicrob Agents Chemother.

[101] Jacobs A, Taccone FS, Roberts JA, Jacobs F, Cotton F, Wolff F (2018). β-Lactam dosage regimens in septic patients with augmented renal clearance. Antimicrob Agents Chemother.

[102] Udy AA, Dulhunty JM, Roberts JA (2017). Association between augmented renal clearance and clinical outcomes in patients receiving β-lactam antibiotic therapy by continuous or intermittent infusion: a nested cohort study of the BLING-II randomised, placebo-controlled, clinical trial. Int J Antimicrob Agents.

[103] Katsube T, Wajima T, Ishibashi T (2016). Pharmacokinetic/pharmacodynamic modeling and simulation of cefiderocol, a parenteral siderophore cephalosporin, for dose adjustment based on renal function. Antimicrob Agents Chemother.

[104] Gaudry S, Hajage D, Schortgen F (2016). Initiation strategies for renal-replacement therapy in the Intensive Care Unit. N Engl J Med.

[105] Crass RL, Rodvold KA, Mueller BA, Pai MP (2019). Renal dosing of antibiotics: are we jumping the gun?. Clin Infect Dis.

[106] Delattre IK, Hites M, Laterre PF (2020). What is the optimal loading dose of broad-spectrum β-lactam antibiotics in septic patients? Results from pharmacokinetic simulation modelling. Int J Antimicrob Agents.

[107] Gatti M, Pea F (2021). Antimicrobial dose reduction in continuous renal replacement therapy: myth or real need? A practical approach for guiding dose optimization of novel antibiotics. Clin Pharmacokinet.

[108] Shields RK, Nguyen MH, Chen L, Press EG, Kreiswirth BN, Clancy CJ (2018). Pneumonia and renal replacement therapy are risk factors for ceftazidime-avibactam treatment failures and resistance among patients with carbapenem-resistant *Enterobacteriaceae* infections. Antimicrob Agents Chemother.

[109] Alobaid AS, Hites M, Lipman J (2016). Effect of obesity on the pharmacokinetics of antimicrobials in critically ill patients: a structured review. Int J Antimicrob Agents.

[110] Hites M, Taccone FS, Wolff F (2013). Case-control study of drug monitoring of β-Lactams in obese critically Ill patients. Antimicrob Agents Chemother.

[111] Meng L, Mui E, Holubar MK, Deresinski SC (2017). Comprehensive guidance for antibiotic dosing in obese adults. Pharmacother J Hum Pharmacol Drug Ther.

[112] Xiao AJ, Huntington JA, Long J, Caro L (2018). Ceftolozane-tazobactam dose regimens in severely-morbidly obese patients with complicated intra-abdominal infection or complicated urinary tract infection. Int J Antimicrob Agents.

[113] Magiorakos AP, Srinivasan A, Carey RB, Carmeli Y, Falagas ME, Giske CG (2012). Multidrug-resistant, extensively drug-resistant and pandrug-resistant bacteria: an international expert proposal for interim standard definitions for acquired resistance. Clin Microbiol Infect.

