# Supplementary material for: The place of new antibiotics for Gram-negative bacterial infections in intensive care: report of a consensus conference
Source: Ann Intensive Care. 2023 Jul 4;13:59. doi: 10.1186/s13613-023-01155-4 (PMC10317910; doi:10.1186/s13613-023-01155-4)
Supplement: Supplementary file 1 — Additional file 1. members of the bibliography group; PICO questions (Patient, Intervention, Comparator, Outcome). [file 13613_2023_1155_MOESM1_ESM.pdf]

# Supplementary Appendix

### *Members of the bibliography group*

Pierrick Bauduin (médecine intensive réanimation, CHU, Caen, France)

Sylvain Le Pape (médecine intensive réanimation, CHU, Poitiers, France)

Lionet Tchatat-Wangueu (médecine intensive réanimation, CHU, Tours, France)

Hélène Messet-Charrière (médecine intensive réanimation, CHU, Tours, France)

Misylias Bouaoud (médecine intensive réanimation, CHU, Tours, France)

Dima Siblani (médecine intensive réanimation, CH, Mulhouse, France).

### *PICO Questions (Patient, Intervention, Comparator, Outcome)*

| 1. Is there a place for the empirical use of the new beta-lactams active against Gram-negative bacteria in the Intensive Care setting? | |
|---|---|
| P | Critically ill patients with clinical suspicion of infection with multidrug-resistant Gram-negative bacteria (BLSE, EPC, ABRI and PARC) |
| I | Use of empirical antibiotic therapy with one of the new beta-lactams (ceftolozane-tazobactam, OR ceftazidime–avibactam OR ceftazidime–avibactam + aztreonam, OR meropenem-vaborbactam, OR imipenem-cilastatin-relebactam, OR cefiderocol |
| C | In comparison with empirical antibiotic therapy using a broad range carbapenem (imipenem-cilastatin OR meropenem) |
| O | Is it associated with a reduction: (i) in mortality rates; (ii) in the length of mechanical ventilation; (iii) in the length of the state of shock; (iv) in the length of stay; (v) of clinical failure; (vi) of the risk of emergence of pan-drug-resistant bacteria? |

| 2. In the context of documented infections with susceptibility to more than one of these antibiotics, is there any pharmacokinetic, pharmacodynamic, ecological, or cost-effectiveness evidence for prioritization? | |
|---|---|
| P | Critically ill patients with a documented Gram-negative bacterial infection |
| I | Use of ceftolozane-tazobactam OR ceftazidime–avibactam OR ceftazidime–avibactam + aztreonam OR meropenem-vaborbactam, OR imipenem-cilastatin-relebactam, OR cefiderocol |
| C | In comparison with the use of ceftolozane-tazobactam OR ceftazidime–avibactam OR ceftazidime–avibactam + aztreonam OR meropenem-vaborbactam, OR imipenem-cilastatin-relebactam, OR cefiderocol |
| O | Is it associated with: (i) a better pharmacokinetic/pharmacodynamic profile; (ii) a lower risk of emergence of multidrug-resistant bacteria; (iii) a lower cost? |

| 3. What are the possible combinations with these antibiotics, and in what context? | |
|---|---|
| P | Critically ill patients with a documented multi-drug-resistant Gram-negative bacterial infection |
| I | Use of ceftolozane-tazobactam OR ceftazidime–avibactam OR ceftazidime–avibactam + aztreonam OR meropenem-vaborbactam, OR imipenem-cilastatin-relebactam, OR cefiderocol |
| C | In comparison with antibiotic therapy combining one of these new antibiotics with another antibiotic (amikacin or colistin or tigecycline) |
| O | Is it associated with a reduction: (i) in mortality rates; (ii) in the length of mechanical ventilation; (iii) in the length of the state of shock; (iv) in the length of stay; (v) of clinical failure; (vi) of the risk of emergence of pan-drug-resistant bacteria? |

| 4. Should these new antibiotics be included in a carbapenem-sparing strategy? | |
|---|---|
| P | Critically ill patients with a documented infection with multidrug-resistant Gram-negative bacteria but carbapenem-susceptible (BLSE, Amp C) |
| I | Use of ceftolozane-tazobactam OR ceftazidime–avibactam OR cefiderocol |
| C | In comparison with antibiotic therapy using carbapenems (imipenem-cilastatin OR meropenem) |
| O | Is it associated with: (i) more survival; (ii) less risk of emergence of multidrug-resistant bacteria: carbapenem-resistant enterobacterales, carbapenem-resistant *Acinetobacter baumani*, carbapenem-resistant *Pseudomonas aeruginosa*; (iii) a lower cost? |

| | |
|---|---|
| 5. What pharmacokinetic and pharmacodynamic data are available in critically ill patients to optimize the mode of administration, particularly continuous infusion, dose increase, and administration strategy guided by measurement of plasma antibiotic concentration? | |
| 5a | |
| P | Critically ill patients |
| I | Continuous infusion (with or without an intravenous bolus injection) of the antibiotic |
| C | Compared to intermittent infusion of the antibiotic |
| O | Is it able to increase the overall time that the target plasma concentration is maintained? |
| 5b | |
| P | Critically ill patients |
| I | Increasing the dose |
| C | Compared to the dosage regimen of a patient not in critical care |
| O | Is it able to increase the overall time that the target plasma concentration is maintained? |
| 5c | |
| P | Critically ill patients |
| I | A strategy of antibiotic administration guided by measurement of plasma levels |
| C | Compared to a strategy without measuring plasma levels |
| O | Is it able to increase the overall time that the target plasma concentration is maintained? |

| 6a. How should doses be adjusted in renal failure? | |
|---|---|
| P | Critically ill patients with an impaired glomerular filtration rate |
| I | Reducing the dose |
| C | Compared to the dosing regimen of a patient with a normal glomerular filtration rate |
| O | Does it reduce the risk of drug toxicity without reducing the overall time that the target plasma concentration is maintained? |
| **6b. How should doses be adjusted in hepatocellular failure?** | |
| P | Critically ill patients with hepatocellular damage |
| I | Reducing the dose |
| C | Compared to the dosage regimen of a patient without hepatocellular damage |
| O | Does it reduce the risk of drug toxicity without reducing the overall time that the target plasma concentration is maintained? |
| **6c. How should doses be adjusted in obesity?** | |
| P | Critically ill obese patients (BMI >= 30 $kg/m^2$) |
| I | Increasing the dose |
| C | Compared to the dosage regimen of a non-obese patient |
| O | Does it increase the overall time that the target plasma concentration is maintained without increasing drug toxicity? |
